# Prediction of cardiac surgery associated acute kidney injury using response to loop diuretic and urine neutrophil gelatinase associated lipocalin

**DOI:** 10.1007/s00467-024-06469-4

**Published:** 2024-08-09

**Authors:** Emily Sullivan, Katherine Melink, Kevin Pettit, Stuart L. Goldstein, Huiayu Zang, Nicholas J. Ollberding, Megan SooHoo, Jeffrey A. Alten, Natalja L. Stanski, Katja M. Gist

**Affiliations:** 1 Department of Pediatrics, Cincinnati Children’s Hospital Medical Center, University of Cincinnati College of Medicine, 3333 Burnet Ave, MLC 2003, Cincinnati, OH 45226 USA; 2 Department of Pediatrics, Children’s Hospital Colorado, University of Colorado Anschutz Medical Campus, Aurora, CO USA

**Keywords:** Acute kidney injury, Urine neutrophil gelatinase associated lipocalin, Loop diuretic, Pediatric cardiac surgery

## Abstract

**Background:**

Cardiac surgery associated acute kidney injury (CS-AKI) is common. Urine response to loop diuretic and urine neutrophil gelatinase associated lipocalin (uNGAL) are separately associated with CS-AKI. We aimed to determine whether urine response to loop diuretic *and* uNGAL together were associated with postoperative day 2–4 CS-AKI.

**Methods:**

Two-center prospective observational study (ages 0–18 years). uNGAL (8–12 h after admission) (ng/mL) and urine response to loop diuretic (6 h for bolus furosemide and 12 h for infusion bumetanide) (mL/kg/hr) were measured. All diuretic doses were converted to furosemide equivalents. The primary outcome was day 2–4 CS-AKI. Patients were sub-phenotyped using a priori cutoffs (uNGAL +  ≥ 100 ng/mL and UOP +  < 1.5 mL/kg/hr) and optimal cutoffs (uNGAL +  ≥ 127 ng/mL and UOP +  ≤ 0.79 mL/kg/hr): 1) uNGAL–/UOP–, 2) uNGAL–/UOP + , 3) uNGAL + /UOP–, and 4) uNGAL + /UOP + . Multivariable regression was used to assess the association of uNGAL, UOP and each sub-phenotype with outcomes.

**Results:**

476 patients were included. CS-AKI occurred in 52 (10.9%). uNGAL was associated with 2.59-fold greater odds (95%CI: 1.52–4.41) of CS-AKI. UOP was not associated with CS-AKI. Compared with uNGAL + alone, uNGAL + /UOP + improved prediction of CS-AKI using a priori and optimal cutoffs respectively (AUC 0.70 vs. 0.75). Both uNGAL + /UOP + (IQR OR:4.63, 95%CI: 1.74–12.32) and uNGAL + /UOP– (IQR OR:5.94, 95%CI: 2.09–16.84) were associated with CS-AKI when compared with uNGAL–/UOP–.

**Conclusions:**

uNGAL is associated with CS-AKI. The sub-phenotype association was largely driven by uNGAL. Future studies standardizing diuretic dose and timing may be needed to refine the combined performance for clinical decision making.

**Graphical abstract:**

**Figure:**
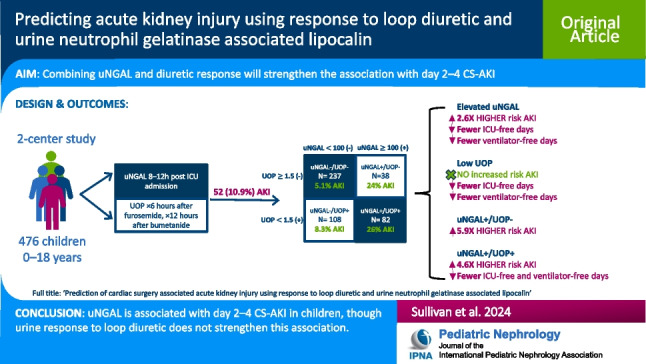
A higher resolution version of the Graphical abstract is available as Supplementary information

**Supplementary Information:**

The online version contains supplementary material available at 10.1007/s00467-024-06469-4.

## Introduction

Acute kidney injury (AKI) is a common, well-documented complication of critical illness in pediatric patients that is associated with poor outcomes, especially when severe. Pediatric cardiac surgery, particularly with cardiopulmonary bypass (CPB), increases risk for developing cardiac surgery associated AKI (CS-AKI) [1, 2]. Children who develop CS-AKI, especially when prolonged or severe, experience prolonged intensive care unit (ICU) and hospital length of stay, prolonged use of mechanical ventilation, and increased mortality [3–7]. Despite the recognized prevalence and consequences of CS-AKI in children following CPB [8], therapies remain limited to supportive care measures that are typically initiated after CS-AKI has been identified. As the limitations of serum creatinine (SCr) are well recognized, current research is directed at earlier recognition of CS-AKI and identification of patients at risk for developing severe disease using novel biomarkers and bedside tools with the goal of allowing earlier, more proactive intervention [9, 10].

A recent report from the neonatal and pediatric heart and renal outcomes network demonstrated that the vast majority of CS-AKI occurred on the first postoperative day [8]. In this study, only stage 3 CS-AKI was associated with mortality but no other clinically relevant outcome. These data highlight the challenges of CS-AKI diagnosis using the current consensus definition, especially given the multitude of factors that can result in elevations of SCr or decrements in urine output (UOP) in the pediatric (particularly neonatal) population. The recent pediatric Acute Disease Quality Initiative (ADQI) highlighted opportunities for improving diagnostic precision among those at risk for AKI by sub-phenotyping patients based on available tools [11]. The authors of this consensus document highlight that multiple sub-phenotypes of AKI can exist in the same patient at the same time. Elevations in urinary biomarkers as well as response to diuretics were called out as specific ways to delineate AKI sub-phenotypes which could have important prognostic and therapeutic implications. Urine neutrophil gelatinase associated lipocalin (uNGAL), a tubular injury biomarker, has been used to predict CS-AKI [12, 13]. Similarly, impaired response to a loop diuretic when given after cardiac surgery has also been associated with development of subsequent CS-AKI [14–16].

Whether combining uNGAL with loop diuretic response improves its association with CS-AKI is unknown. In this study, we aimed to determine whether uNGAL measured within 8–12 h after cardiac surgery, coupled with response to a loop diuretic, improved the association with day 2–4 CS-AKI. We hypothesized that combining uNGAL and diuretic response would strengthen the association with day 2–4 CS-AKI.

## Methods

We performed a secondary analysis of a 2-center prospective observational study of children aged 0–18 years undergoing cardiac surgery from two sources: 1) Children who were enrolled in the Heart Institute biorepository at Cincinnati Children’s Hospital Medical Center (CCHMC) and 2) Children at Children’s Hospital Colorado who had samples collected from a related prospective observational study. Children who had urine samples collected for the study, a pre-operative SCr and more than one SCr on postoperative days 2–4 were included. Exclusion criteria were pre-existing kidney disease within 72 h of enrollment, kidney failure defined as an estimated glomerular filtration rate (eGFR) < 30 mL/min/1.73 m^2^, and cardiac arrest or use of extracorporeal life support prior to the index surgery [17]. Institutional review board approval was obtained at each site and included a waiver of informed consent (Children’s Hospital Colorado). This manuscript follows the Strengthening the Reporting of Observational Studies in Epidemiology (STROBE) guidelines (Supplemental Table 1).

Demographic, clinical and outcome data were extracted from the pediatric cardiac critical care consortium (PC4) database at each site and merged into a single de-identified dataset. Clinical data not available in PC4 were manually extracted from the electronic medical record. uNGAL was collected 8–12 h after ICU admission and on the first postoperative morning. UOP was quantified hourly in response to the first dose of loop diuretic, either furosemide or bumetanide, after ICU admission. Urine was quantified and indexed to weight and hour for the first 6 h after furosemide and 12 h after bumetanide (administered as a continuous infusion). Twelve hours was chosen for bumetanide based on pharmacist recommendation (Personal communication: Dr. Melanie Joy, PharmD, PhD) for 2 reasons: 1) the volume and infusion rate often take several hours to “reach” the patient after the infusion is initiated, and 2) the time to reach steady state exceeds 8 h in neonates and children [18, 19]. Total bumetanide dose for 6 h was converted to furosemide equivalents with 1 mg bumetanide = 40 mg furosemide [19].

### Definitions

CS-AKI was defined using the Kidney Disease: Improving Global Outcomes (KDIGO) SCr criteria [17]. Urine output was not used to diagnose AKI given the small number of patients with indwelling bladder catheters beyond the first postoperative day. Loop diuretic responsiveness was defined as UOP ≥ 1.5 mL/kg/hr for the first 6 h after furosemide or 12 h for those receiving a bumetanide infusion. This cutoff was chosen a priori based on the spread of data as well as the previously published literature [15, 16]. Lack of response to diuretic is classified as UOP + . A positive uNGAL was defined as ≥ 100 ng/mL. This was chosen based on the relatively low concentration among the entire cohort (upper quartile of 110 ng/mL). Using these cutoffs, patients were also sub-phenotyped based on each of the individual variables as follows: 1) uNGAL–/UOP– 2) uNGAL–/UOP + , 3) uNGAL + /UOP–, and 4) uNGAL + /UOP + . Surgical complexity was defined using the Society of Thoracic Surgeons—European Association for Cardio-Thoracic Surgery Congenital Heart Surgery (STAT) Mortality Categories [20].

The primary outcome was postoperative day 2–4 CS-AKI. Secondary outcomes were 28-day ventilator-free days, 28-day ICU-free days and 28-day mortality [21]. Ventilator and ICU-free days were zero for patients who died. Patients who received peritoneal drainage or prophylactic peritoneal dialysis were not classified as having AKI or having received kidney replacement therapy (KRT) [8].

### Statistical analysis

Continuous variables were reported as medians with interquartile ranges (IQR) and compared using Wilcoxon rank-sum tests or Kruskal–Wallis tests as appropriate. Categorical variables were reported as frequencies with percentages and compared using chi-square tests.

Multivariable logistic regression was used to develop a prediction model for day 2–4 CS-AKI. Model predictors were selected a priori based on the existing literature and clinical practice and included indexed UOP (mL/kg/hr), uNGAL (ng/mL), age (months), STAT category (> 3 vs. ≤ 3), single ventricle status, CPB duration (mins), furosemide dose equivalents (mg/kg), and delayed sternal closure. The indexed UOP and uNGAL were flexibly modeled using restricted cubic spline with three knots placed at the 10th, 50th and 90th percentiles as recommended by Harrell [22]. The interaction between indexed UOP and uNGAL was assessed using a likelihood ratio test (LRT). Given the lack of significance for interacting effect (LRT *p* = 0.597), we opted to remove it for model parsimony. Estimates for the adjusted effects of predictors on the odds of day 2–4 CS-AKI were reported as odds ratios (OR) and the corresponding 95% confidence intervals (CI) using Wald statistics. For continuous predictors, the ORs were rescaled to reflect an increase from the first (25th percentile) to the third quartile (75th percentile); known as IQR OR. To evaluate additive predictive risk of a biomarker for day 2–4 CS-AKI, we reported the concordance statistic (c-statistic), Brier score, Nagelkerke R square, Akaike information criterion, adequacy index (log-likelihood base model/log-likelihood new model), and conducted LRT for model comparison [22]. Internal validation of discrimination ability was performed using bootstrap resampling to provide optimism-corrected results.

A separate logistic regression model was used to evaluate the association of day 2–4 CS-AKI status with mortality. The ordinal regression models were fitted for 28-day ventilator-free days and 28-day ICU-free days. The ordinal regression model was used as it is advantageous for skewed outcome variables while preserving power [23]. For continuous secondary outcomes estimated via the ordinal regression model, a common OR was calculated, where an OR < 1.0 indicated a factor associated with increased healthcare utilization (i.e., fewer ventilator and ICU-free days).

Similar modeling approaches were used to determine the association of outcomes with uNGAL and UOP sub-phenotypes as defined above. In addition, we examined sub-phenotypes derived by selecting thresholds for uNGAL and UOP that individually maximized Youden’s index (i.e., balanced sensitivity and specificity). A two-sided *p*-value of < 0.05 was considered statistically significant. All analyses were performed using R version 4.3.1 statistical software. The rms package (version 6.7.1) was used to perform regression and model validation. The cut point r-package (version 1.1.2) was used to derive the optimal cut points for uNGAL and UOP.

## Results

After exclusions (missing uNGAL and missing SCr [*n* = 115]), 476 children were included in the analysis (Supplemental Fig. 1). Median age (IQR) among them was 4.77 (1.43–30.40) months. Four hundred forty-four patients (94%) underwent CPB, 151 (32%) had single ventricle status, and there were 23 (4.8%) deaths. The median (IQR) number of ventilator-free days was 27 (25, 28) and ICU-free days was 23 (15, 26).

### Postoperative day 2–4 CS-AKI associations

Fifty-two (10.9%) patients developed day 2–4 CS-AKI (Supplemental Fig. 1), of which 32 (6.7%) were classified as severe (stage 2 or 3) and 11 (2.3%) received KRT. Table 1 summarizes the demographics, clinical features, and outcomes of patients with day 2–4 CS-AKI compared to those without. There was no difference in age (as a continuous variable) and sex among patients with and without day 2–4 CS-AKI. There was no difference in furosemide dose equivalents indexed to weight between groups. Patients with day 2–4 CS-AKI had higher surgical complexity as evidenced by a higher proportion of STAT 4 or 5 operations, more frequent single ventricle heart disease (46% vs. 30%), and longer CPB duration (180 min, [IQR: 123–284] vs. 146 min [IQR: 105–197] compared to those with no day 2–4 CS-AKI (*p* = 0.007, *p* = 0.027, and *p* = 0.006 respectively). Patients with day 2–4 CS-AKI had a higher severity of illness as measured by vasoactive inotrope score at 8 h after CICU admission (*p* = 0.005). Patients with day 2–4 CS-AKI had a median of 1 less ventilator-free day and 8 less ICU-free days compared to those without day 2–4 CS-AKI (*p* = 0.018 and *p* < 0.001 respectively). Mortality was 4 times higher among patients with day 2–4 CS-AKI compared to no day 2–4 CS-AKI (15% vs. 3.5%, *p* < 0.001).

**Table 1:** Demographics, clinical features, and outcomes of those with and without AKI

Variable	Overall (*N* = 476)	No CS-AKI (*N* = 424)	Day 2–4 CS-AKI (*N* = 52)	*p*-value
Age (months)	4.77 (1.43, 30.40)	4.58 (1.40, 28.14)	9.11 (2.04, 51.82)	0.09
Age < 1 year	319 (67%)	292 (69%)	27 (52%)	**0.022**
Sex category				0.90
Female	185 (39%)	166 (40%)	19 (37%)	
Male	286 (61%)	254 (60%)	32 (63%)	
STAT category				**0.007**
1	80 (17%)	80 (19%)	0 (0%)	
2	133 (29%)	119 (29%)	14 (28%)	
3	59 (13%)	53 (13%)	6 (12%)	
4	146 (31%)	123 (30%)	23 (46%)	
5	48 (10%)	41 (9.9%)	7 (14%)	
Single ventricle	151 (32%)	127 (30%)	24 (46%)	**0.027**
Weight at surgery (kg)	5.82 (3.90, 13.00)	5.67 (3.90, 12.72)	8.20 (3.95, 16.08)	0.14
Cardiopulmonary bypass	444 (94%)	395 (94%)	49 (96%)	0.80
Cardiopulmonary bypass duration (min)	148.00 (107.75, 201.25)	146.00 (105.50, 197.00)	180.00 (123.00, 284.00)	**0.006**
Circ arrest (min)	43.00 (0.00, 89.00)	43.00 (0.00, 85.75)	52.00 (0.00, 148.50)	0.20
x-clamp (min)	78.00 (38.25, 127.50)	76.00 (37.50, 122.00)	110.00 (54.00, 187.00)	**0.016**
Delayed sternal closure	77 (16%)	59 (14%)	18 (35%)	** < 0.001**
VIS-8Hour	8 (5, 12)	7 (5, 11)	10 (7, 14)	**0.005**
uNGAL (ng/mL)	25.95 (10.00, 110.50)	24.30 (10.00, 76.62)	214.50 (21.73, 496.25)	** < 0.001**
Furosemide dose equivalents (mg/kg)	0.99 (0.70, 1.10)	0.99 (0.70, 1.08)	0.99 (0.64, 1.11)	0.80
Indexed urine output (ml/kg/hr)	1.95 (0.95, 3.73)	2.10 (1.03, 3.75)	1.10 (0.46, 2.54)	** < 0.001**
UOP < 1 ml/kg	125 (27%)	100 (24%)	25 (49%)	** < 0.001**
Day 2–4 severe AKI	32 (6.7%)	0 (0%)	32 (62%)	** < 0.001**
CKRT	11 (2.3%)	0 (0%)	11 (21%)	** < 0.001**
Ventilation duration (days)	1 (0, 3)	1 (0, 2)	2 (0, 4)	**0.014**
ICU LOS (days)	4 (2, 12)	4 (2, 11)	12 (4, 21)	** < 0.001**
Ventilator-free days	27 (25, 28)	27 (25, 28)	26 (23, 28)	**0.018**
ICU-free days	23 (15, 26)	24 (17, 26)	16 (3, 22)	** < 0.001**
Death	23 (4.8%)	15 (3.5%)	8 (15%)	** < 0.001**

*AKI* acute kidney injury, *STAT* Society of Thoracic Surgeons-European Association for Cardio-Thoracic Surgery, *circ* circulatory, *VIS* vasoactive-inotropic score, *uNGAL* urine neutrophil gelatinase associated lipocalin, *UOP* urine output, *CKRT* continuous kidney replacement therapy, *ICU* intensive care unit, *LOS* length of stay. Ventilator duration and ICU LOS is among survivors only

### Loop diuretic comparisons

Furosemide was the most common diuretic used in 60% (*n* = 281). Supplemental Table 2 compares the demographics, clinical features and outcomes of those who received bumetanide vs. furosemide. Those who received bumetanide were younger, underwent more complex surgeries and were sicker than those who received furosemide. Day 2–4 CS-AKI occurred in 16% (*n* = 30) of those who received bumetanide and 7.5% (*n* = 21) of those who received furosemide (*p* = 0.005). There was no difference in mortality between groups, but those exposed to bumetanide had fewer ventilator and ICU-free days.

### Associations of continuous UOP response and uNGAL with postoperative day 2–4 CS-AKI

**Fig. 1:**
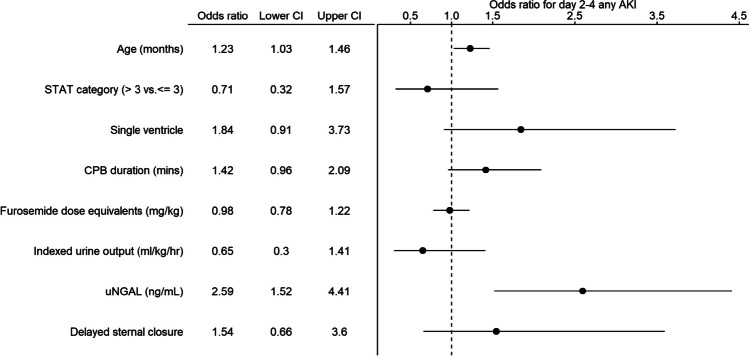
Forest plot of predictors of day 2–4 acute kidney injury (AKI). Odds ratio (OR) and 95% confidence interval (CI) from a multivariable logistic regression model. For continuous variables, ORs were rescaled to reflect an increase from the first (25th percentile) to the third quartile (75th percentile). *STAT*: Society of Thoracic Surgeons-European Association for Cardio-Thoracic Surgery, *CBP*: cardiopulmonary bypass, *uNGAL*: urine neutrophil gelatinase associated lipocalin

**Fig. 2:**
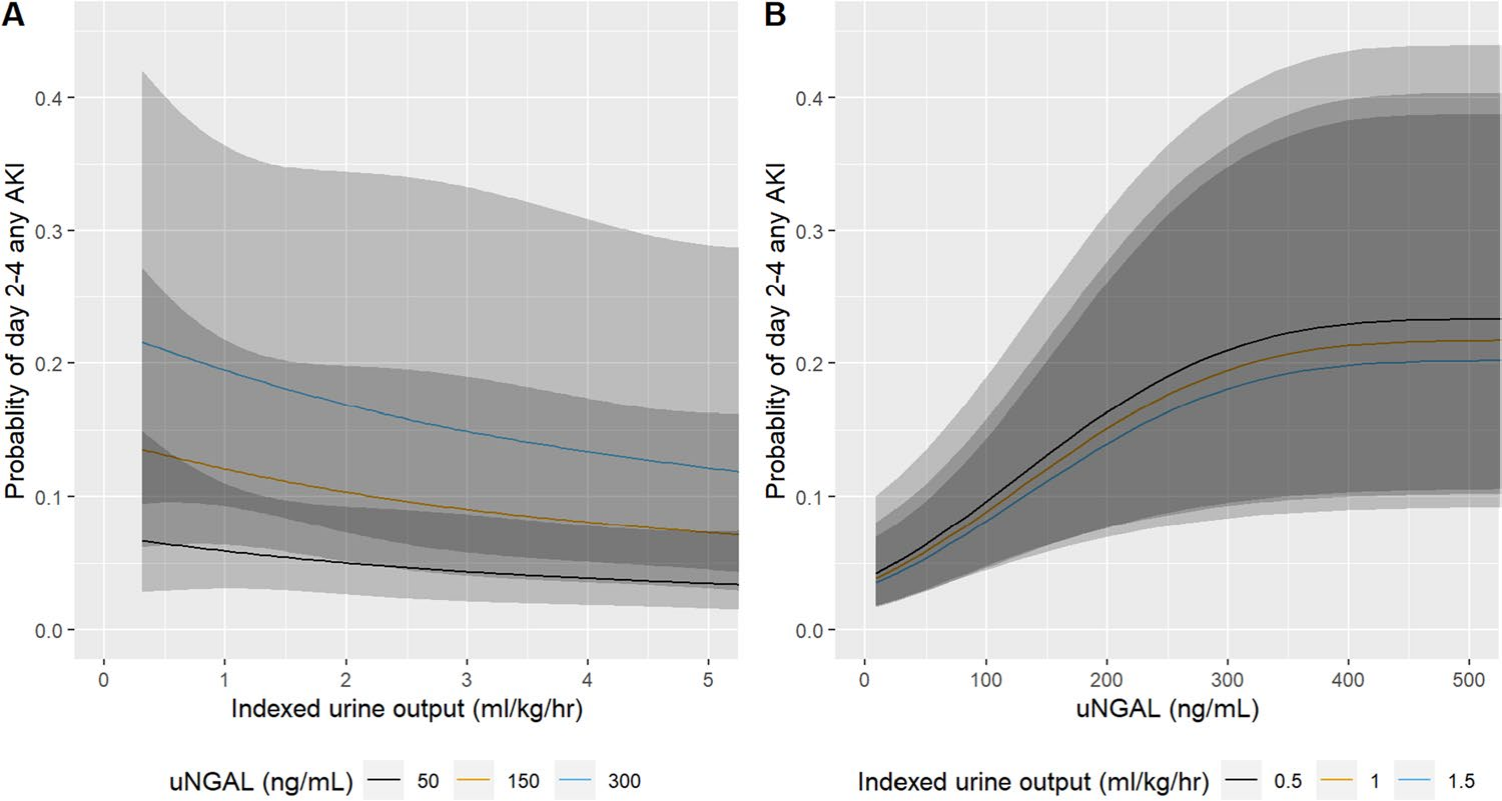
Probability of day 2–4 acute kidney injury (AKI) as a function of indexed urine output (**A**) and urine neutrophil gelatinase associated lipocalin (uNGAL) (**B**) from multivariable logistic regression, adjusted for a priori relevant covariates at the most frequent or median level. Indexed urine output and uNGAL were modeled with restricted cubic splines (3 knots) to allow for potential non-linear association with day 2–4 any AKI. Shaded area denotes 95% confidence intervals

Patients with day 2–4 CS-AKI had higher uNGAL (214.50 [IQR: 21.73–496.25] vs. 24.30 [10.00–76.62]; *p* < 0.001) and lower indexed UOP in response to loop diuretic (1.10 [0.46–2.54] vs. 2.10 [1.03–3.75]; *p* < 0.001). In multivariable logistic regression, uNGAL modeled as a continuous variable was associated with 2.59 greater odds of day 2–4 CS-AKI when comparing a value of 110.5 ng/mL (75th percentile) to a value of 10.0 ng/mL (25th percentile), IQ OR: 2.59 [1.52–4.41]) (Figs. 1, and 2B). Indexed UOP alone was not associated with day 2–4 CS-AKI (OR: 0.65 [0.30–1.41]) (Fig. 1). The predicted probabilities for day 2–4 CS-AKI when modeled as a non-linear function of uNGAL and UOP are presented in Fig. 2. The interaction term of uNGAL and UOP on the risk of day 2–4 CS-AKI was not significant (LRT chi-square = 1.88, degree of freedom [df] = 3, *p* = 0.597, Supplemental Table 3).

**Fig. 3:**
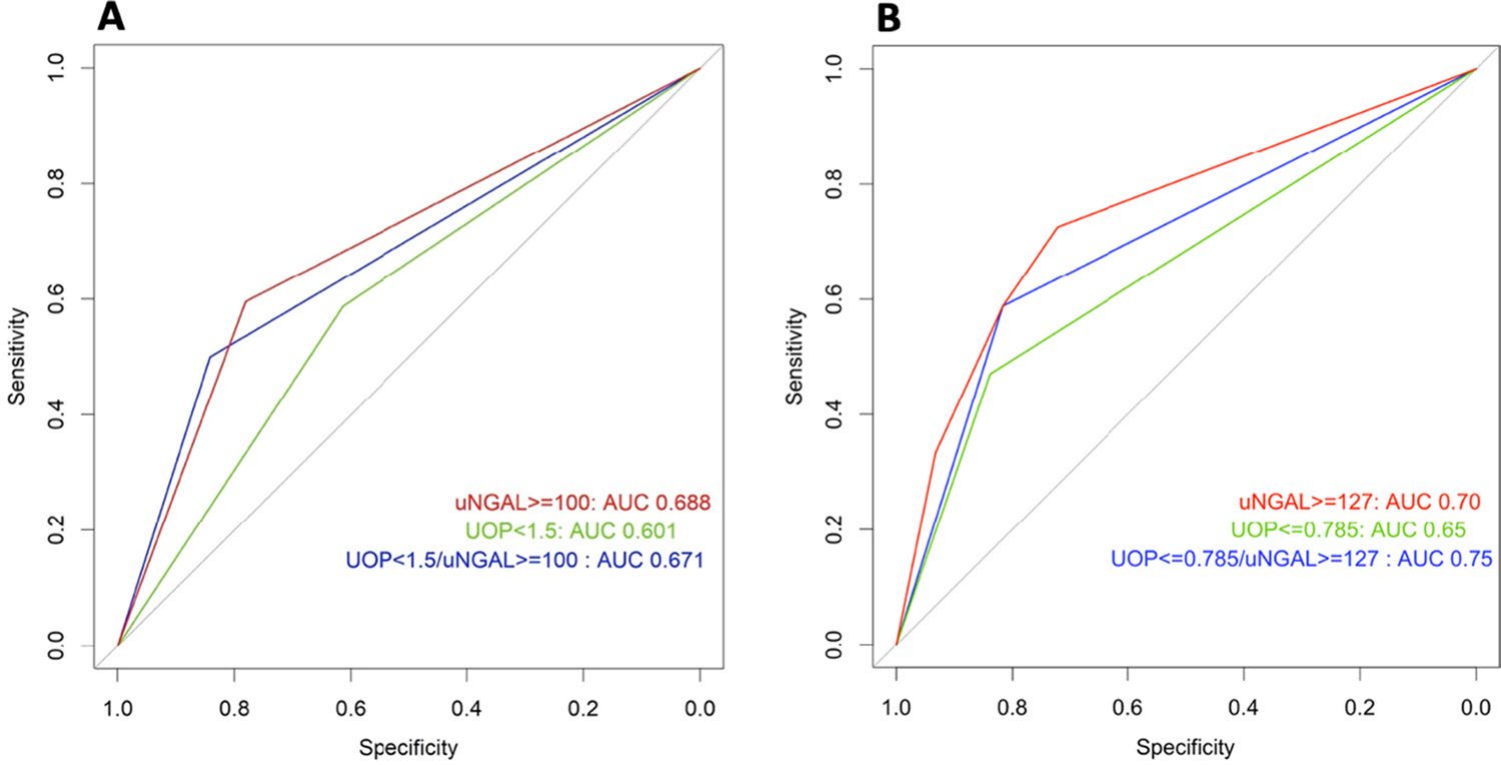
Observed receiver operating curves for prediction of day 2–4 AKI using a priori cutoffs (**A**) vs optimal cutoffs defined by Youden’s index (**B**). Area under the receiving operator curve (AUC) for prediction of postoperative day 2–4 AKI using uNGAL ≥ 100 ng/mL alone, UOP < 1.5 mL/kg/h alone, and UOP < 1.5 mL/kg/h + uNGAL ≥ 100 ng/mL (A). AUC for prediction of postoperative day 2–4 AKI using uNGAL ≥ 127 ng/mL alone, UOP ≤ 0.79 mL/kg/h alone, and UOP ≤ 0.79 mL/kg/h + NGAL ≥ 127 ng/mL (B). *UOP*: urine output, *uNGAL*: urine neutrophil gelatinase associated lipocalin

We compared uNGAL alone and UOP alone for prediction of day 2–4 CS-AKI using univariate receiver operating curve analysis. The area under the receiver operating curve (AUC) using a priori cutoffs for uNGAL and UOP for predicting day 2–4 CS-AKI was 0.69 and 0.60, respectively (Fig. 3A). We then sought to determine the most optimal cutoffs for uNGAL and UOP based on Youden’s index. The optimal cutoff for uNGAL was ≥ 127 ng/mL and for UOP was ≤ 0.79 mL/kg/hr. The AUC for each was 0.70 and 0.65, respectively, for predicting day 2–4 CS-AKI (Fig. 3B). The sensitivity, specificity, positive predictive value (PPV) and negative predictive value (NPV) using both the a priori and optimal cutoffs are summarized in Supplemental Table 4.

Comparing c-statistics of multivariable logistic regression, the models with uNGAL alone, uNGAL + UOP, and uNGAL + UOP + their interaction had similar performance for prediction of day 2–4 CS-AKI (optimism-corrected c-statistics: 0.744 vs. 0.737 vs. 0.722) (Supplemental Table 3). The adequacy index comparing likelihood ratios for the model with uNGAL only to that with uNGAL + UOP was 0.966. UOP adds only a fraction of 0.034 of new predictive information to uNGAL. The model LRT shows that the addition of UOP to uNGAL did not significantly improve discrimination for predicting day 2–4 CS-AKI (LRT chi-square = 1.65, df = 2, *p* = 0.439) (Supplemental Table 4).

Because of the non-linear relationship between uNGAL and UOP with day 2–4 CS-AKI, we assessed the probability of day 2–4 CS-AKI for each predictor individually but stratified by the other (Fig. 2). The probability of day 2–4 CS-AKI increases with lower UOP when stratified by uNGAL concentration (Fig. 2A). There was a decrease in UOP with higher thresholds of uNGAL. The probability of day 2–4 CS-AKI also increases with increasing uNGAL at different thresholds of UOP (Fig. 2B). There was a significant increase in uNGAL with lower thresholds of UOP. 

### Associations with secondary outcomes

In multivariable analysis, higher uNGAL was associated with fewer ventilator-free (IQR OR: 0.59, 95%CI: 0.44–0.79) and fewer ICU-free days (IQR OR: 0.72, 95%CI: 0.53–0.96) (Supplemental Fig. 2A, B). uNGAL was not associated with mortality (IQR OR: 1.46, 95%CI: 0.65–3.29) (Supplemental Fig. 2C). Higher indexed UOP was associated with more ventilator-free (IQR OR: 3.51, 95%CI: 2.31–5.33) and more ICU-free (IQR OR: 2.09, 95%CI: 1.38–3.15) days (Supplemental Fig. 2D, E), but not mortality (IQR OR: 0.80, 95%CI: 0.23–2.72) (Supplemental Fig. 2F).

### Sub-phenotypes of uNGAL and urine output

Of the 4 sub-phenotypes (Supplemental Fig. 3): 237 patients (51%) uNGAL–/UOP–, 108 (23%) uNGAL–/UOP + , 38 (8%) uNGAL + /UOP–, and 82 (18%) uNGAL + /UOP + . Demographics, clinical characteristics and outcomes for the sub-phenotypes are summarized in Table 2. Those with uNGAL + /UOP + were significantly younger than all other sub-phenotypes with a median (IQR) age of 0.38 months (0.20, 3.17) and 91% (*n* = 75) being < 1 year (*p* < 0.001). There were also significant differences in intraoperative and postoperative characteristics across sub-phenotypes, with those with uNGAL + /UOP + having the highest surgical complexity, longer circulatory arrest, increased utilization of delayed sternal closure and higher vasoactive inotrope score at 8 h as compared to other sub-phenotypes (Table 2).

**Table 2:** Demographics, clinical features, and outcomes across the 4 sub-phenotypes

Variable	Overall, *N* = 465	NGAL and UOP cutoff				*p*-value^*2*^
		uNGAL–/UOP– *N* = 237^*1*^	uNGAL–/UOP + *N* = 108^*1*^	uNGAL + /UOP– *N* = 38^*1*^	uNGAL + /UOP + *N* = 82^*1*^	
Age (months)	4.77 (1.53, 30.33)	7.87 (3.77, 43.00)	4.49 (2.07, 14.93)	3.41 (0.43, 16.59)	0.38 (0.20, 3.17)	** < 0.001**
Age < 1 year	312 (67%)	131 (55%)	79 (73%)	27 (71%)	75 (91%)	** < 0.001**
Sex category						0.60
Female	183 (40%)	99 (42%)	43 (41%)	12 (32%)	29 (36%)	
Male	277 (60%)	137 (58%)	63 (59%)	26 (68%)	51 (64%)	
STAT category						** < 0.001**
1	78 (17%)	58 (25%)	12 (12%)	4 (11%)	4 (4%)	
2	128 (28%)	89 (38%)	27 (26%)	6 (16%)	6 (7%)	
3	59 (13%)	27 (11%)	19 (18%)	3 (8%)	10 (12%)	
4	144 (32%)	57 (24%)	34 (33%)	17 (46%)	36 (44%)	
5	47 (10%)	4 (2%)	11 (11%)	7 (19%)	25 (31%)	
Single ventricle	150 (32%)	77 (32%)	25 (23%)	13 (34%)	35 (43%)	**0.041**
Weight at surgery (kg)	5.80 (3.93, 13.00)	7.59 (4.91, 14.90)	5.47 (3.97, 9.00)	4.70 (3.51, 7.68)	3.83 (3.16, 4.87)	** < 0.001**
Cardiopulmonary bypass	437 (95%)	215 (91%)	106 (100%)	37 (97%)	79 (99%)	**0.002**
Cardiopulmonary bypass duration (min)	148.00 (107.00, 200.00)	132.00 (100.00, 175.00)	163.50 (113.25, 219.50)	171.00 (122.00, 225.00)	170.00 (137.50, 231.00)	** < 0.001**
Circ arrest (min)	43.50 (0.00, 89.00)	29.00 (0.00, 80.75)	47.50 (0.00, 102.00)	36.50 (9.50, 77.00)	73.00 (23.75, 105.50)	** < 0.001**
x-clamp (min)	79.00 (40.00, 127.00)	70.00 (24.50, 114.50)	99.00 (55.25, 151.75)	73.00 (30.00, 130.25)	84.50 (53.75, 143.50)	** < 0.001**
Delayed sternal closure	74 (16%)	11 (5%)	16 (15%)	6 (16%)	41 (51%)	** < 0.001**
VIS-8Hour	8 (5, 12)	5 (5, 9)	9 (7, 15)	8 (7, 12)	11 (8, 15)	** < 0.001**
uNGAL (ng/mL)	25.70 (10.00, 107.00)	11.60 (10.00, 26.90)	25.10 (11.88, 49.62)	233.50 (131.50, 442.00)	397.00 (186.50, 986.75)	** < 0.001**
Furosemide dose equivalents (mg/kg)	1.00 (0.72, 1.10)	0.99 (0.70, 1.00)	1.01 (0.70, 1.40)	1.00 (0.95, 1.14)	1.10 (0.80, 1.30)	** < 0.001**
Indexed urine output (ml/kg/hr)	1.95 (0.95, 3.73)	3.36 (2.43, 5.01)	0.92 (0.65, 1.26)	2.92 (2.14, 5.17)	0.69 (0.55, 1.04)	** < 0.001**
UOP < 1 ml/kg	125 (27%)	0 (0%)	65 (60%)	0 (0%)	60 (73%)	** < 0.001**
Day 2–4 any AKI	51 (11%)	12 (5.1%)	9 (8.3%)	9 (24%)	21 (26%)	** < 0.001**
Day 2–4 severe AKI	31 (6.7%)	9 (3.8%)	6 (5.6%)	4 (11%)	12 (15%)	**0.006**
CKRT	10 (2.2%)	7 (3.0%)	1 (0.9%)	1 (2.6%)	1 (1.2%)	0.60
Death	22 (4.7%)	5 (2.1%)	3 (2.8%)	3 (7.9%)	11 (13%)	** < 0.001**
Ventilation duration (days)	1 (0, 3)	1 (0, 1)	1 (0, 3)	1 (1, 3)	3 (2, 6)	** < 0.001**
ICU LOS (days)	4 (2, 12)	3 (2, 6)	6 (3, 14)	8 (3, 20)	14 (8, 23)	** < 0.001**
Ventilator-free days	27 (25, 28)	27 (27, 28)	27 (25, 28)	27 (24, 27)	24 (20, 26)	** < 0.001**
ICU-free days	23 (16, 26)	25 (22, 26)	21 (13, 25)	19 (7, 25)	13 (2, 20)	** < 0.001**

^*1*^ Statistics presented: median (IQR); n (%)

^*2*^Statistical tests performed: Kruskal–Wallis test; chi-square test of independence

uNGAL + defined as ≥ 100 ng/mL, UOP + defined as quantified urine output < 1.5 mL/kg/hr

*uNGAL* urine neutrophil gelatinase associated lipocalin, *UOP* urine output, *STAT* Society of Thoracic Surgeons-European Association for Cardio-Thoracic Surgery, *circ* circulatory, *VIS* vasoactive-inotropic score, *AKI* acute kidney injury, *CKRT* continuous kidney replacement therapy, *ICU* intensive care unit, *LOS* length of stay. Ventilator duration and ICU LOS is among survivors only

#### Associations of uNGAL and UOP sub-phenotypes with postoperative day 2–4 CS-AKI

**Fig. 4:**
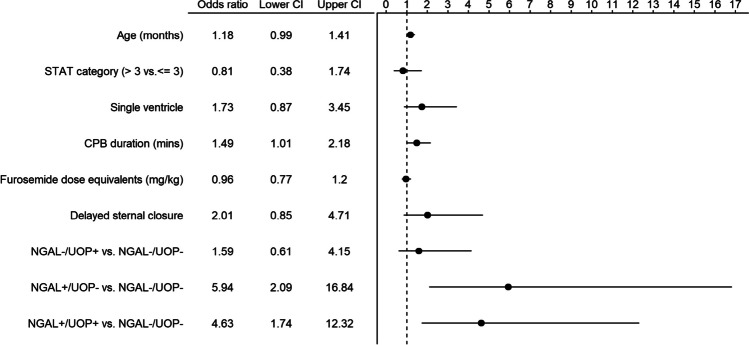
Forest plot of sub-phenotypes as predictors of day 2–4 AKI. Odds ratio (OR) and 95% confidence interval (CI) from a multivariable logistic regression model including the predictors listed in the figure. For continuous variables, ORs were rescaled to reflect an increase from the first (25th percentile) to the third quartile (75th percentile). Both uNGAL + /UOP + and uNGAL + /UOP– are associated with day 2–4 AKI when compared with uNGAL–/UOP–

The frequency of day 2–4 CS-AKI was highest among the uNGAL + /UOP + sub-phenotype (26%, *n* = 21) followed by uNGAL + /UOP– (24%, *n* = 9) (*p* < 0.001). In multivariable logistic regression, both uNGAL + /UOP + (IQR OR: 4.63, 95%CI: 0.65–3.29) and uNGAL + /UOP– (IQR OR: 5.94, 95%CI: 2.09–16.84) were associated with day 2–4 CS-AKI when compared with uNGAL–/UOP–, after adjusting for covariates (Fig. 4). The test characteristics (sensitivity, specificity, PPV and NPV) for each of the sub-phenotypes using the a priori cutoffs and the optimal cutoffs are summarized in Supplemental Table 4. Using the optimal cutoffs (uNGAL ≥ 127 and UOP ≤ 0.79), the AUC for uNGAL + /UOP + improved from 0.67 to 0.75 (Fig. 3).

#### Associations of uNGAL and UOP sub-phenotypes with secondary outcomes

The uNGAL + /UOP + sub-phenotype had the fewest ventilator-free days (24, IQR: 20–26) (*p* < 0.001). The uNGAL + /UOP + sub-phenotype had the fewest ICU-free days (13, IQR: 2–20) followed by the uNGAL + /UOP– group (19, IQR: 7–25) (*p* < 0.001). Mortality was significantly higher in those with uNGAL + /UOP + as compared to all the other sub-phenotypes (*p* < 0.001).

When compared with the uNGAL–/UOP– sub-phenotype using logistic regression and ordinal regression models, the uNGAL + /UOP + group had fewer ventilator-free days (OR: 0.17, 95%CI: 0.10–0.29) and fewer ICU-free days (OR: 0.31, 95%CI: 0.18–0.53) (Supplemental Fig. 4). There was no association of uNGAL + /UOP + with mortality (OR:3.25, 95%CI: 0.74–14.33) (Supplemental Fig. 4).

## Discussion

In this 2-center study of children undergoing cardiac surgery, uNGAL, as in other reports, is predictive of postoperative day 2–4 CS-AKI, but UOP in response to a loop diuretic is not. Using a priori cutoffs, uNGAL with UOP in response to loop diuretic did not enhance the predictive performance of day 2–4 CS-AKI. However, after optimizing cutoffs, the predictive value of uNGAL with urine response to loop diuretic for day 2–4 CS-AKI improved with a better balance in sensitivity and specificity as well as improvement in NPV. As such, uNGAL in combination with UOP response to diuretic may be useful for risk stratifying children susceptible to CS-AKI. To better understand the utility of these tests for clinical decision support, diuretic dose and timing should be standardized in future studies.

Considering recent data demonstrating the challenges in CS-AKI diagnosis using the current consensus definition, structural biomarkers like uNGAL, which was recently approved by the Food and Drug Administration for use in critically ill children aged 3 months to 21 years, may play an important role in delineating AKI risk and guiding management in children after cardiac surgery and in other populations of critically ill children. uNGAL is specific to kidney tubular damage, rises quickly and rises in proportion to the degree of injury [24]. In prior studies of patients after CPB, uNGAL has been associated with earlier identification of subsequent SCr-defined CS-AKI, AKI severity and clinical prognosis [13, 25–27]. We demonstrate, as in previous work, that an early rise in uNGAL after CPB is associated with non-transient, clinically significant AKI, although in our study, we measure uNGAL much later than in prior reports [28]. Despite this, uNGAL can be used in real-time at the bedside to guide decision-making around fluid management and initiation of KRT in this population.

Urine response to loop diuretic has been reported in multiple retrospective pediatric cardiac surgery studies to be associated with AKI development [14–16]. However, in our study, indexed UOP alone was not associated with day 2–4 CS-AKI. This could be explained by several factors. First, we defined CS-AKI as occurring on postoperative days 2–4, which likely reflects persistent rather than transient disease directly related to cardiac surgery. In addition, although all patients in our study received diuretics before day 2, the dose and timing of furosemide or initiation of and dose of bumetanide were not standardized across patients. In the published studies, the time frame in which AKI could be diagnosed extended from the first through sixth postoperative day, and the timing of furosemide and UOP response relative to AKI diagnosis may overlap. In addition, this protracted time frame likely represents different AKI sub-phenotypes that cannot be compared directly to the current study. We also included patients who received a bumetanide infusion, and immediate assessment of response may be more complex than how it was handled in this study. We measured UOP for 12 h for patients who received a continuous bumetanide infusion to account for the time to reach steady state, but since the half-life of bumetanide is variable based on patient age, this timing may have been suboptimal for some patients [29]. To date, there are no prospective studies evaluating response to diuretics using a standardized time and dose for pediatric CS-AKI prediction.

Given the heterogeneous nature of pediatric AKI, expert consensus supports the use of sub-phenotypes to refine risk assessment and guide therapy [11]. The use of factors including urine biomarkers and response to loop diuretic to characterize dynamic sub-phenotypes over time may refine AKI diagnosis and direct clinical management, particularly after cardiac surgery. Indeed, stratification of patients using the renal angina index for clinical decision support for personalization of fluid management and KRT initiation was recently described for non-cardiac surgery related critical illness [30, 31]. Goldstein and colleagues reported that renal angina index-directed biomarker measurement (uNGAL and furosemide response) resulted in reduced fluid overload, earlier time to KRT initiation, shorter ICU length of stay and improved survival to ICU discharge [30]. While we collected uNGAL and furosemide response to loop diuretic for all patients meeting inclusion criteria regardless of CS-AKI risk in this study, we have future work similarly focused on who should receive this testing after cardiac surgery. AKI diagnosis is especially challenging in children after cardiac surgery due to the use of intraoperative ultrafiltration, fluid shifts that occur during and after CPB, and altered perfusion associated with congenital heart lesions, which make traditional markers of kidney function unreliable [32]. We demonstrate that sub-phenotyping patients by uNGAL and urine response to loop diuretic provides insight to day 2–4 CS-AKI risk and may augment clinical decision making. However, as is seen in our data, the association between the sub-phenotypes and day 2–4 CS-AKI is largely driven by uNGAL.

These data can be used for clinical decision support at the bedside as they reflect the degree of kidney injury in real time. While there are currently no prevention or treatment options for CS-AKI, early recognition prior to the injury becoming irreversible provides the opportunity to optimize supportive care, including initiating KRT in a timely fashion [30]. While uNGAL predicts CS-AKI within hours of injury to the kidney, furosemide response cannot be assessed until the patient demonstrates acceptable hemodynamics. As such, early risk stratification with uNGAL may guide management including vasoactive and fluid administration and timing and choice of access in the immediate post-operative period, allowing prompt initiation of KRT by the time hemodynamic stability is achieved and functional kidney injury is determined [33].

### Study limitations

The strength of this study is that it included two centers with varying clinical practice patterns. There are, however, several limitations. Urine response to loop diuretic was collected retrospectively, thus the dose and timing of loop diuretic after cardiac surgery was not standardized. In addition, the incidence of day 2–4 CS-AKI in our study population (10%) was low in comparison to other studies and emphasizes that the majority of elevation of SCr after cardiac surgery resolves in the first 24 h and may not represent tubular dysfunction [3]. Of note, nearly 20% of patients from our initial study population were excluded for missing SCr or missing uNGAL, possibly biasing toward a sicker patient population that would have had these assessments done more frequently. In addition, deriving optimal cutpoints and assessing model performance on the same set of patient values may have contributed to optimistic estimates.

## Conclusions

uNGAL is associated with day 2–4 CS-AKI after pediatric cardiac surgery. In our study, urine response to loop diuretic was not independently associated with day 2–4 CS-AKI, though it may strengthen the association of uNGAL with CS-AKI when sub-phenotyping patients based on both predictors. Both elevated uNGAL and low UOP in response to loop diuretic were associated with fewer ICU-free and fewer ventilator-free days. Prospective studies are needed to further examine the utility of sub-phenotyping for refining CS-AKI risk assessment in children after cardiac surgery.

## Supplementary Information

Below is the link to the electronic supplementary material.

Graphical abstract (PPTX 95 KB)

Supplemental Figures and Tables (DOCX 585 KB)

## Data Availability

The datasets generated during and/or analyzed during the current study are available from the corresponding author on reasonable request.
