# Supplementary material for: Prediction of cardiac surgery associated acute kidney injury using response to loop diuretic and urine neutrophil gelatinase associated lipocalin
Source: Pediatr Nephrol. 2024 Aug 9;39(12):3597–606. doi: 10.1007/s00467-024-06469-4 (PMC11511769; doi:10.1007/s00467-024-06469-4)
Supplement: Supplementary file 1 — Graphical abstract (PPTX 95 KB) [file 467_2024_6469_MOESM1_ESM.pptx]

## Slide 1
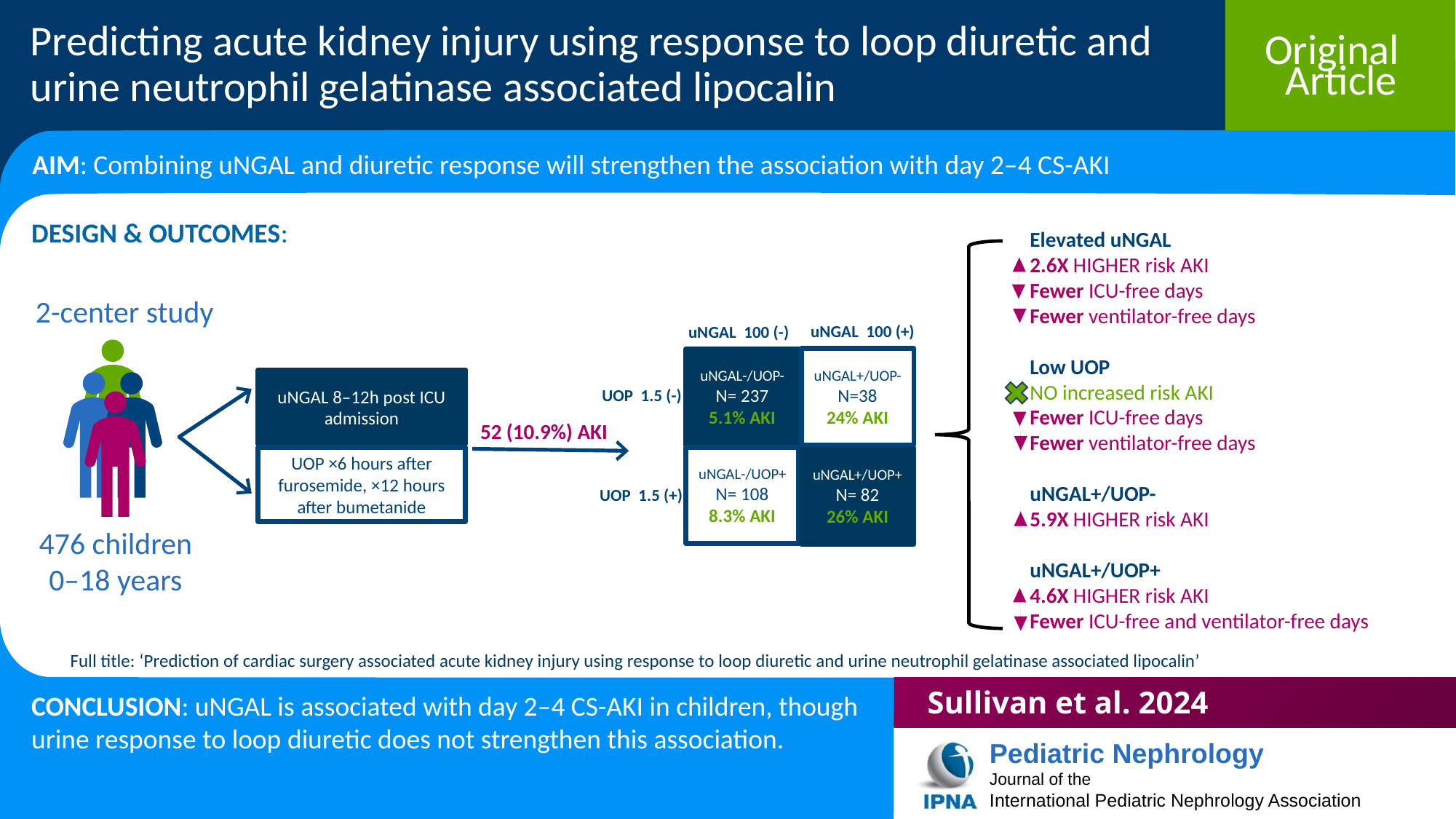

Predicting acute kidney injury using response to loop diuretic and urine neutrophil gelatinase associated lipocalin
AIM: Combining uNGAL and diuretic response will strengthen the association with day 2–4 CS-AKI
DESIGN & OUTCOMES:
Elevated uNGAL
2.6X HIGHER risk AKI
Fewer ICU-free days
Fewer ventilator-free days
Low UOP
NO increased risk AKI
Fewer ICU-free days
Fewer ventilator-free days
uNGAL+/UOP-
5.9X HIGHER risk AKI
uNGAL+/UOP+
4.6X HIGHER risk AKI
Fewer ICU-free and ventilator-free days
2-center study
uNGAL+/UOP-
N=38
24% AKI
uNGAL-/UOP-
N= 237
5.1% AKI
uNGAL 8–12h post ICU admission
52 (10.9%) AKI
uNGAL-/UOP+
N= 108
8.3% AKI
UOP ×6 hours after furosemide, ×12 hours after bumetanide
uNGAL+/UOP+
N= 82
26% AKI
476 children
0–18 years
Full title: ‘Prediction of cardiac surgery associated acute kidney injury using response to loop diuretic and urine neutrophil gelatinase associated lipocalin’
Sullivan et al. 2024
CONCLUSION: uNGAL is associated with day 2–4 CS-AKI in children, though urine response to loop diuretic does not strengthen this association.
