# Supplementary material for: Prediction of cardiac surgery associated acute kidney injury using response to loop diuretic and urine neutrophil gelatinase associated lipocalin
Source: Pediatr Nephrol. 2024 Aug 9;39(12):3597–606. doi: 10.1007/s00467-024-06469-4 (PMC11511769; doi:10.1007/s00467-024-06469-4)
Supplement: Supplementary file 2 — Supplemental Figures and Tables (DOCX 585 KB) [file 467_2024_6469_MOESM2_ESM.docx]

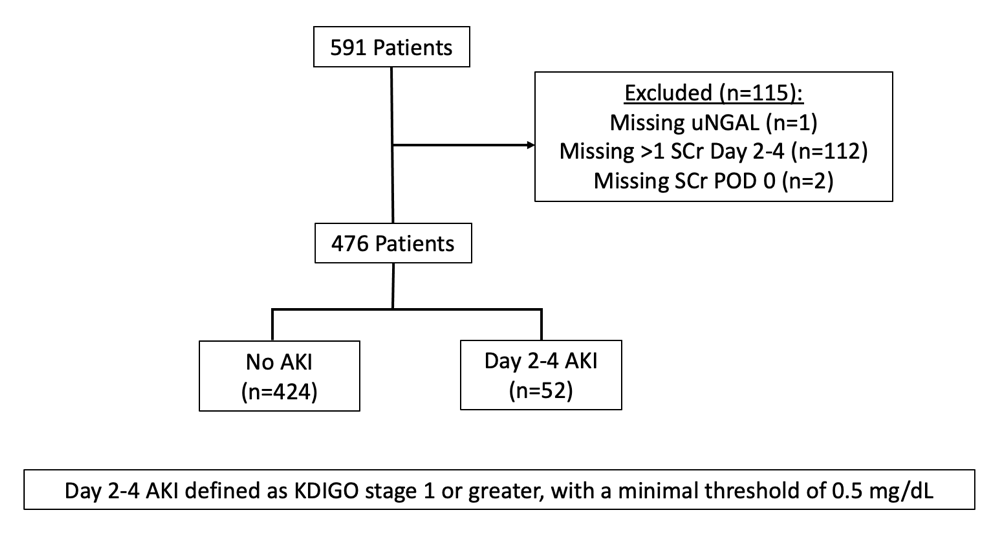


**Supplemental Figure 1. Consort Flow diagram.** After exclusions for missing urine neutrophil gelatinase associated lipocalin (uNGAL) and missing serum creatinine (SCr), 476 patients were included. Of those included, 52 (10.9%) developed post-operative day 2-4 AKI by Kidney Disease: Improving global outcomes SCr criteria.


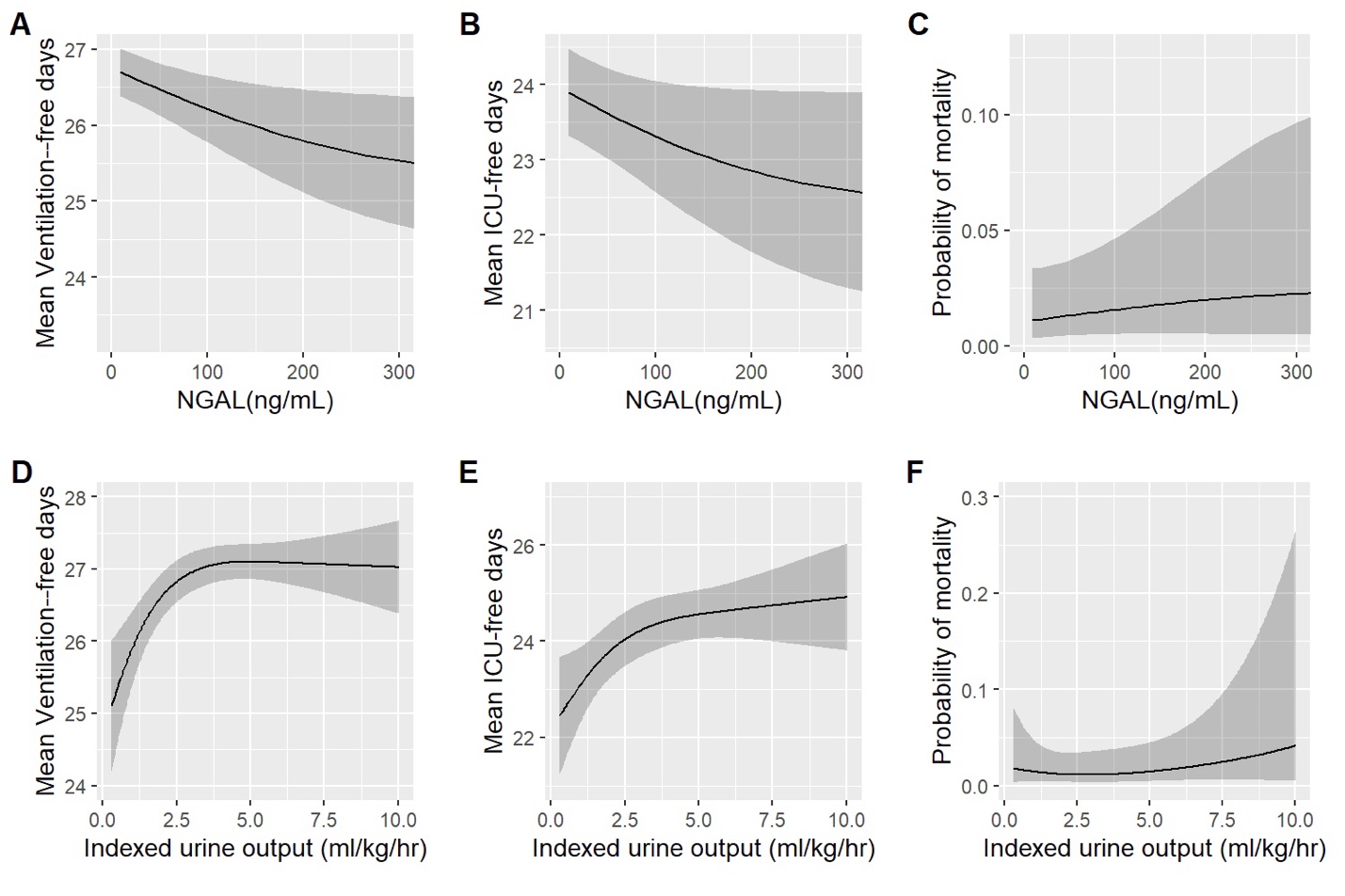


**Supplemental Figure 2. Predicted clinical secondary outcomes (28-day ventilator free days, 28-day ICU free days and 28-day mortality) as a function of urine neutrophil gelatinase associated lipocalin (uNGAL) (A-C) and indexed urine output (UOP)(D-F) from logistic regression and ordinal regression models.** Indexed UOP and uNGAL were modeled with restricted cubic splines (3 knots) to allow for potential non-linear association with secondary outcomes. Shaded area denotes 95% confidence intervals. Higher uNGAL is associated with fewer ventilator-free days (IQR OR:0.59, 95%CI: 0.44-0.79) (A) and fewer ICU-free days (IQR OR:0.72, 95%CI: 0.53-0.96) (B). uNGAL is not associated with mortality (C). Higher indexed UOP is associated with more ventilator-free days (IQR OR:3.51, 95%CI: 2.31-5.33) (D) and more ICU-free days (IQR OR:2.09, 95%CI: 1.38-3.15) (E). Indexed UOP is not associated with mortality (F).

**
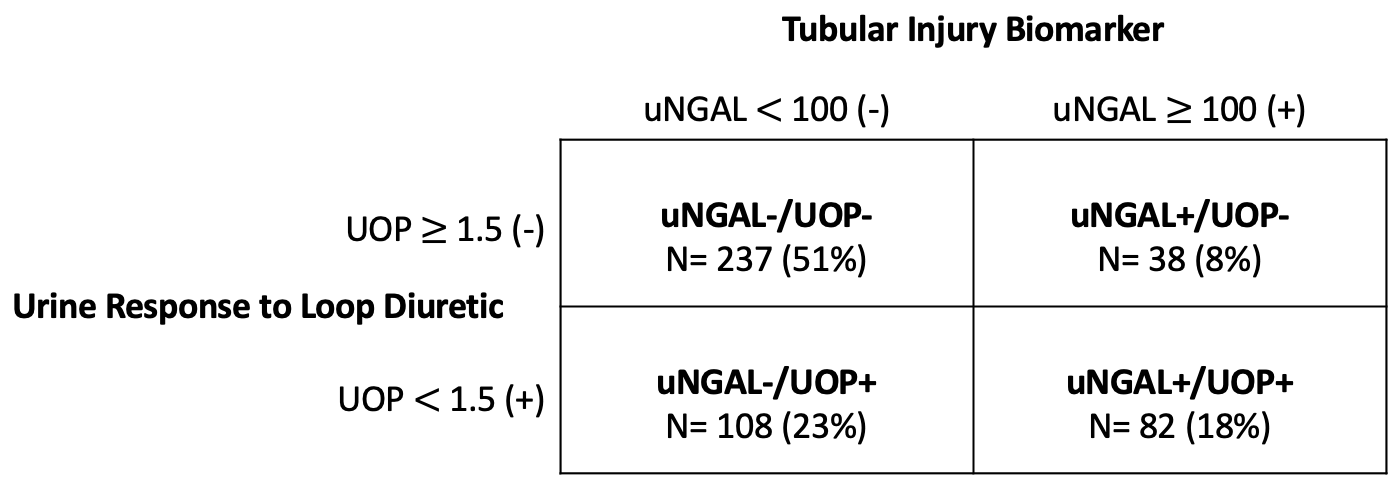
**

**Supplemental Figure 3. Sub-phenotypes based on uNGAL and UOP.** Number and percentage of patients designated to each sub-phenotype. Urine neutrophil gelatinase associated lipocalin (uNGAL) is measured in ng/mL. Urine output (UOP) in response to loop diuretic is measured in mL/kg/h. Plus sign denotes abnormal value.

**
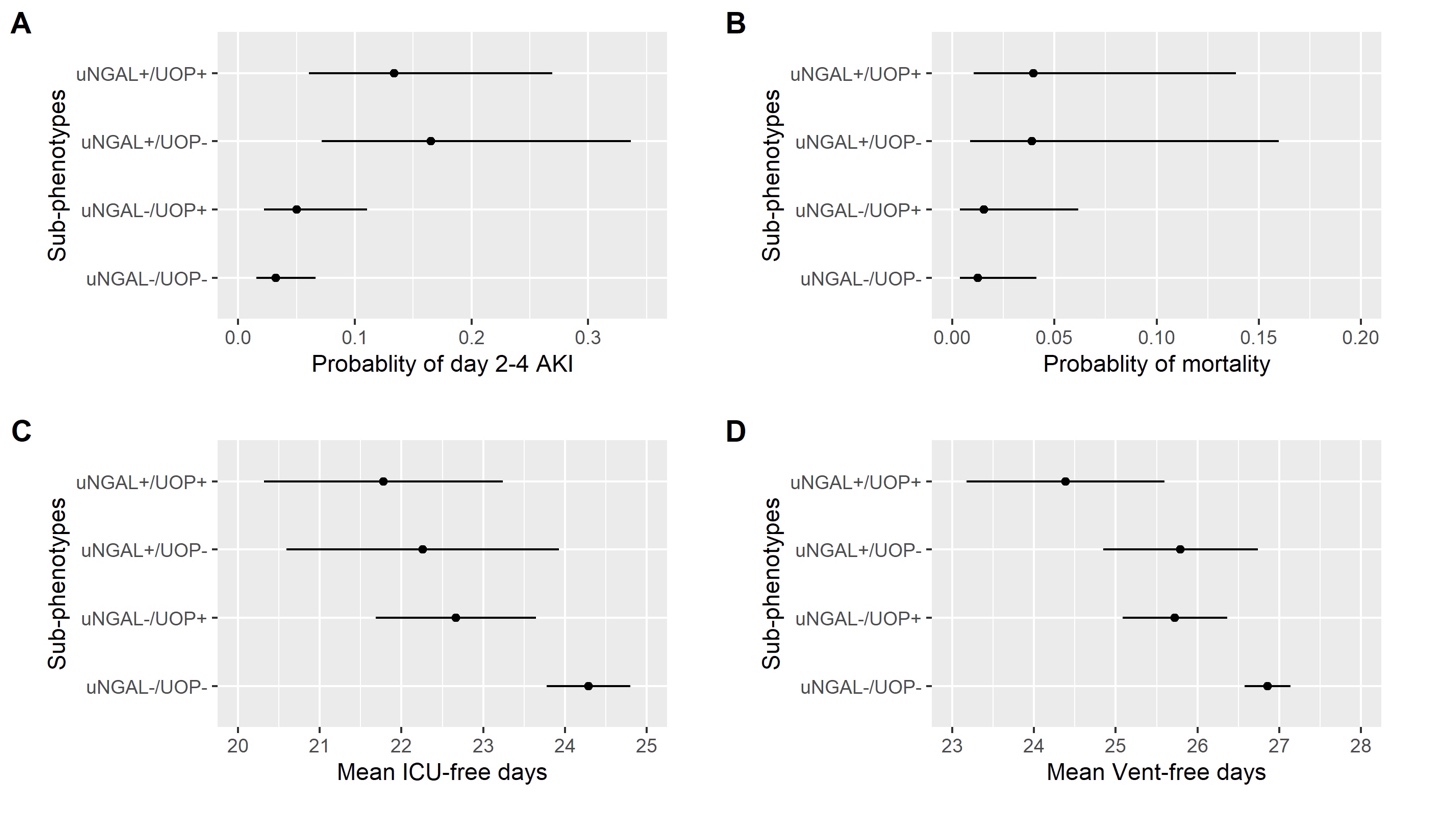
**

**Supplemental Figure 4. Predicted clinical outcomes of day 2-4 acute kidney injury (AKI) (A), mortality (B), intensive care unit (ICU)-free days (C) and ventilator-free days (D) by sub-phenotypes from logistic regression and ordinal regression models.** All probabilities or means are adjusted to the most frequent or median level of remaining covariates included in the model. Both uNGAL+/UOP+ (IQR OR:4.63, 95%CI: 0.65-3.29) and uNGAL+/UOP- (IQR OR:5.94, 95%CI: 2.09-16.84) are associated with day 2-4 AKI when compared with uNGAL-/UOP-. Comparing with uNGAL-/UOP-, uNGAL+/UOP+ had fewer ICU-free (OR:0.31, 95%CI: 0.18-0.53) and ventilator-free days (OR:0.17, 95%CI: 0.10-0.29), but no statistically significant difference in mortality (OR:3.25, 95%CI: 0.74-14.33).

| **Supplemental Table 1. Strobe Checklist. Prediction of cardiac surgery associated acute kidney injury using response to loop diuretic and urine neutrophil gelatinase associated lipocalin.**   \|  \| Item No \| Recommendation \| Page \| \| --- \| --- \| --- \| --- \| \| **Title and abstract** \| 1 \| (*a*) Indicate the study’s design with a commonly used term in the title or the abstract \| 1 \| \| (*b*) Provide in the abstract an informative and balanced summary of what was done and what was found \| 2 \| \| Introduction \| \| \|  \| \| Background/rationale \| 2 \| Explain the scientific background and rationale for the investigation being reported \| 3, 4 \| \| Objectives \| 3 \| State specific objectives, including any prespecified hypotheses \| 4 \| \| Methods \| \| \|  \| \| Study design \| 4 \| Present key elements of study design early in the paper \| 4, 5 \| \| Setting \| 5 \| Describe the setting, locations, and relevant dates, including periods of recruitment, exposure, follow-up, and data collection \| 4, 5 \| \| Participants \| 6 \| (*a*) *Cohort study*—Give the eligibility criteria, and the sources and methods of selection of participants. Describe methods of follow-up \| 4 \| \| (*b*) *Cohort study*—For matched studies, give matching criteria and number of exposed and unexposed \| n/a \| \| Variables \| 7 \| Clearly define all outcomes, exposures, predictors, potential confounders, and effect modifiers. Give diagnostic criteria, if applicable \| 5, 6 \| \| Data sources/ measurement \| 8 \| For each variable of interest, give sources of data and details of methods of assessment (measurement). Describe comparability of assessment methods if there is more than one group \| 5, 6 \| \| Bias \| 9 \| Describe any efforts to address potential sources of bias \| 5, 15 \| \| Study size \| 10 \| Explain how the study size was arrived at \| 8 \| \| Quantitative variables \| 11 \| Explain how quantitative variables were handled in the analyses. If applicable, describe which groupings were chosen and why \| 5, 6 \| \| Statistical methods \| 12 \| (*a*) Describe all statistical methods, including those used to control for confounding \| 6, 7 \| \| (*b*) Describe any methods used to examine subgroups and interactions \| 6, 7 \| \| (*c*) Explain how missing data were addressed \| 8 \| \| (*d*) *Cohort study*—If applicable, explain how loss to follow-up was addressed \| n/a \| \| € Describe any sensitivity analyses \| n/a \|   Continued on next page   \| Results \| \| \|  \| \| --- \| --- \| --- \| --- \| \| Participants \| 13 \| (a) Report numbers of individuals at each stage of study—eg numbers potentially eligible, examined for eligibility, confirmed eligible, included in the study, completing follow-up, and analysed \| 4, 8 \| \| (b) Give reasons for non-participation at each stage \| 8 \| \| (c) Consider use of a flow diagram \| Supplemental Figure 1 \| \| Descriptive data \| 14 \| (a) Give characteristics of study participants (eg demographic, clinical, social) and information on exposures and potential confounders \| 8, Table 1 \| \| (b) Indicate number of participants with missing data for each variable of interest \| 8 \| \| (c) *Cohort study*—Summarise follow-up time (eg, average and total amount) \| n/a \| \| Outcome data \| 15 \| *Cohort study*—Report numbers of outcome events or summary measures over time \| 8 \| \| Main results \| 16 \| (*a*) Give unadjusted estimates and, if applicable, confounder-adjusted estimates and their precision (eg, 95% confidence interval). Make clear which confounders were adjusted for and why they were included \| 8-11 \| \| (*b*) Report category boundaries when continuous variables were categorized \| 8-11 \| \| (*c*) If relevant, consider translating estimates of relative risk into absolute risk for a meaningful time period \| n/a \| \| Other analyses \| 17 \| Report other analyses done—eg analyses of subgroups and interactions, and sensitivity analyses \| 9-11 \| \| Discussion \| \| \|  \| \| Key results \| 18 \| Summarise key results with reference to study objectives \| 12 \| \| Limitations \| 19 \| Discuss limitations of the study, taking into account sources of potential bias or imprecision. Discuss both direction and magnitude of any potential bias \| 14, 15 \| \| Interpretation \| 20 \| Give a cautious overall interpretation of results considering objectives, limitations, multiplicity of analyses, results from similar studies, and other relevant evidence \| 12-15 \| \| Generalisability \| 21 \| Discuss the generalisability (external validity) of the study results \| 12 \| \| Other information \| \| \|  \| \| Funding \| 22 \| Give the source of funding and the role of the funders for the present study and, if applicable, for the original study on which the present article is based \| 1 \|   **Note:** An Explanation and Elaboration article discusses each checklist item and gives methodological background and published examples of transparent reporting. The STROBE checklist is best used in conjunction with this article (freely available on the Web sites of PLoS Medicine at http://www.plosmedicine.org/, Annals of Internal Medicine at http://www.annals.org/, and Epidemiology at http://www.epidem.com/). Information on the STROBE Initiative is available at www.strobe-statement.org.  **Supplemental Table 2.** Demographics, clinical features, and outcomes of those who received furosemide vs bumetanide. | | | | |
| --- | --- | --- | --- | --- | --- | --- | --- | --- | --- | --- | --- | --- | --- | --- | --- | --- | --- | --- | --- | --- | --- | --- | --- | --- | --- | --- | --- | --- | --- | --- | --- | --- | --- | --- | --- | --- | --- | --- | --- | --- | --- | --- | --- | --- | --- | --- | --- | --- | --- | --- | --- | --- | --- | --- | --- | --- | --- | --- | --- | --- | --- | --- | --- | --- | --- | --- | --- | --- | --- | --- | --- | --- | --- | --- | --- | --- | --- | --- | --- | --- | --- | --- | --- | --- | --- | --- | --- | --- | --- | --- | --- | --- | --- | --- | --- | --- | --- | --- | --- | --- | --- | --- | --- | --- | --- | --- | --- | --- | --- | --- | --- | --- | --- | --- | --- | --- | --- | --- | --- | --- | --- | --- | --- | --- | --- | --- | --- | --- | --- | --- | --- | --- | --- | --- | --- | --- | --- | --- | --- | --- |
| **Variable** | **Overall**, N = 467*^1^* | Diuretic type | | **p-value***^2^* |
|  |  | **Furosemide**, N = 281*^1^* | **Bumetanide**, N = 186*^1^* |  |
| **Age (months)** | 4.77 (1.58, 30.69) | 7.87 (3.73, 42.87) | 2.00 (0.23, 5.85) | **<0.001** |
| **Age < 1 year** | 312 (67%) | 157 (56%) | 155 (83%) | **<0.001** |
| **Sex** |  |  |  | 0.90 |
| Female | 183 (40%) | 109 (39%) | 74 (40%) |  |
| Male | 279 (60%) | 169 (61%) | 110 (60%) |  |
| **STAT category** |  |  |  | **<0.001** |
| 1 | 79 (17%) | 69 (25%) | 10 (5.6%) |  |
| 2 | 129 (28%) | 104 (37%) | 25 (14%) |  |
| 3 | 59 (13%) | 32 (12%) | 27 (15%) |  |
| 4 | 144 (31%) | 67 (24%) | 77 (43%) |  |
| 5 | 47 (10%) | 6 (2.2%) | 41 (23%) |  |
| **Single ventricle** | 150 (32%) | 78 (28%) | 72 (39%) | **0.017** |
| **Weight at surgery (kg)** | 5.84 (3.94, 13.05) | 6.94 (4.90, 14.60) | 4.07 (3.26, 6.31) | **<0.001** |
| **Cardiopulmonary bypass** | 439 (95%) | 258 (93%) | 181 (98%) | **0.018** |
| **Cardiopulmonary bypass duration (min)** | 148.00 (106.50, 200.50) | 129.50 (100.25, 181.00) | 170.00 (135.00, 227.00) | **<0.001** |
| **Circ arrest (min)** | 44.00 (0.00, 89.25) | 25.00 (0.00, 79.00) | 64.50 (18.00, 110.00) | **<0.001** |
| **x-clamp (min)** | 79.00 (40.00, 128.00) | 70.00 (27.00, 119.00) | 91.00 (60.00, 142.75) | **<0.001** |
| **Delayed sternal closure** | 74 (16%) | 11 (4.0%) | 63 (34%) | **<0.001** |
| **VIS-8Hour** | 7.50 (5.00, 12.00) | 5.50 (5.00, 8.00) | 10.75 (8.00, 16.00) | **<0.001** |
| **Average UOP hr 0-8** | 1.65 (1.22, 2.34) | 1.62 (1.23, 2.18) | 1.73 (1.17, 2.59) | 0.19 |
| **uNGAL (ng/mL)** | 25.70 (10.00, 106.50) | 15.30 (10.00, 39.50) | 86.45 (24.70, 345.75) | **<0.001** |
| **Furosemide dose equivalents (mg/kg)** | 1.00 (0.71, 1.10) | 0.98 (0.66, 1.00) | 1.20 (0.80, 1.60) | **<0.001** |
| **Average hourly UOP indexed (ml/kg)** | 14.33 (4.67, 41.83) | 26.33 (14.25, 56.83) | 3.96 (2.42, 7.48) | **<0.001** |
| **Indexed urine output (ml/kg/hr)** | 1.95 (0.95, 3.73) | 3.20 (2.00, 5.01) | 0.90 (0.61, 1.39) | **<0.001** |
| **UOP < 1ml/kg** | 125 (27%) | 18 (6.5%) | 107 (58%) | **<0.001** |
| **Day 2-4 any AKI** | 51 (11%) | 21 (7.5%) | 30 (16%) | **0.005** |
| **Day 2-4 severe AKI** | 31 (6.6%) | 14 (5.0%) | 17 (9.1%) | 0.11 |
| **CKRT** | 10 (2.1%) | 9 (3.2%) | 1 (0.5%) | 0.10 |
| **Death** | 22 (4.7%) | 9 (3.2%) | 13 (7.0%) | 0.10 |
| **Ventilation duration (days)** | 1.00 (0.14, 2.76) | 0.42 (0.00, 1.00) | 2.90 (1.22, 4.79) | **<0.001** |
| **ICU LOS (days)** | 4.15 (2.00, 11.95) | 2.87 (1.87, 5.00) | 11.97 (5.80, 19.91) | **<0.001** |
| **Ventilator-free days** | 27.00 (25.10, 27.82) | 27.57 (27.00, 28.00) | 25.06 (23.00, 26.55) | **<0.001** |
| **ICU-free days** | 23.36 (15.96, 26.00) | 25.13 (22.94, 26.13) | 16.00 (7.63, 22.11) | **<0.001** |
| *^1^*Median (IQR); n (%); *^2^*Wilcoxon rank sum test; Pearson’s Chi-squared test; STAT: Society of Thoracic Surgeons-European Association for Cardio-Thoracic Surgery, circ: circulatory, VIS: vasoactive-inotropic score, UOP: urine output, uNGAL: urine neutrophil gelatinase associated lipocalin, AKI: acute kidney injury, CKRT: continuous kidney replacement therapy, ICU: intensive care unit, LOS: length of stay. Ventilator duration and ICU LOS is among survivors only. | | | | |

atinase-associated lipocalin, UOP: urine output.

**Supplemental Table 3.** Statistical indices for the additive value of a biomarker for prediction of day 2-4 AKI.

| **Statistical indices** | **uNGAL only** | **uNGAL + UOP** | **uNGAL + UOP + their interaction** |
| --- | --- | --- | --- |
| Apparent C-statistic | 0.782 | 0.787 | 0.791 |
| Optimism-corrected c-statistic | 0.744 | 0.737 | 0.722 |
| Apparent Brier score | 0.087 | 0.086 | 0.084 |
| Optimism-corrected Brier score | 0.093 | 0.094 | 0.095 |
| Apparent R square | 0.197 | 0.207 | 0.215 |
| Optimism-corrected R square | 0.131 | 0.122 | 0.085 |
| AIC | 280.35 | 276.89 | 281.00 |
| Adequacy index |  | 0.966 | 0.962 |
| LRT p value |  | 0.439 | 0.597 |

**We compared the multivariable model with uNGAL only, one with uNGAL + UOP, and one with uNGAL + UOP + their interaction.**All multivariable models were adjusted for pre-specified clinically relevant covariates (Figure 1). The optimism-corrected values were obtained from bootstrap resampling (1000 samples). AIC, Akaike information criterion; LRT, likelihood ratio test.

**Supplemental Table 4**. Test Characteristics for uNGAL, UOP and sub-phenotypes using different cutoffs of uNGAL and UOP for predicting Day 2-4 CS-AKI.

| Contrast | Sensitivity | Specificity | Positive predictive value | Negative predictive value |
| --- | --- | --- | --- | --- |
| A Priori Cutoffs |  |  |  |  |
| uNGAL+ | 0.59 (0.45; 0.71) | 0.78 (0.74; 0.82) | 0.25 (0.18; 0.33) | 0.94 (0.91; 0.96) |
| UOP+ | 0.59 (0.45; 0.71) | 0.61 (0.57; 0.66) | 0.16 (0.11; 0.22) | 0.92 (0.89; 0.95) |
| uNGAL-/UOP+ vs. uNGAL-/UOP- | 0.43 (0.24; 0.63) | 0.69 (0.64; 0.74) | 0.08 (0.04; 0.15) | 0.95 (0.91; 0.97) |
| uNGAL+/UOP- vs. uNGAL-/UOP- | 0.43 (0.24; 0.63) | 0.89 (0.84; 0.92) | 0.24 (0.13; 0.39) | 0.95 (0.91; 0.97) |
| uNGAL+/UOP+ vs. uNGAL-/UOP- | 0.64 (0.47; 0.78) | 0.79 (0.74; 0.83) | 0.26 (0.17; 0.36) | 0.95 (0.91; 0.97) |
| Optimal Cutoffs |  |  |  |  |
| uNGAL+ | 0.59 (0.45; 0.71) | 0.82 (0.78; 0.85) | 0.28 (0.21; 0.38) | 0.94 (0.91; 0.96) |
| UOP+ | 0.47 (0.34; 0.6) | 0.84 (0.8; 0.87) | 0.26 (0.18; 0.36) | 0.93 (0.9; 0.95) |
| uNGAL-/UOP+ vs. uNGAL-/UOP- | 0.33 (0.17; 0.55) | 0.88 (0.85; 0.91) | 0.15 (0.08; 0.28) | 0.96 (0.93; 0.97) |
| uNGAL+/UOP- vs. uNGAL-/UOP- | 0.48 (0.31; 0.66) | 0.86 (0.82; 0.89) | 0.21 (0.13; 0.33) | 0.96 (0.93; 0.97) |
| uNGAL+/UOP+ vs. uNGAL-/UOP- | 0.55 (0.38; 0.71) | 0.91 (0.88; 0.94) | 0.38 (0.25; 0.52) | 0.96 (0.93; 0.97) |

Optimal cutoffs: determined using Youden’s method, uNGAL+ is >127 ng/mL and UOP+ is ≤0.79 mL/kg/h. A priori cutoffs: uNGAL+ is >100 ng/mL and UOP+ is <1.5 mL/kg/h. uNGAL: urine neutrophil gel
